# Aqueous Humor Cytokines in Non-Proliferative Diabetic Retinopathy

**DOI:** 10.3390/medicina58070909

**Published:** 2022-07-08

**Authors:** Otilia Obadă, Anca Delia Pantalon, Gabriela Rusu-Zota, Anca Hăisan, Smaranda Ioana Lupuşoru, Daniela Constantinescu, Dorin Chiseliţă

**Affiliations:** 1 Department of Ophthalmology, “Grigore T. Popa” University of Medicine and Pharmacy, 16 Universităţii Street, 700115 Iaşi, Romania; chiselita.dorin@gmail.com; 2 Department of Ophthalmology, “Saint Spiridon” Clinical Emergency Hospital, 1 Independenţei Street, 700111 Iaşi, Romania; anca_pantalon@yahoo.com; 3 Department of Pharmacology, “Grigore T. Popa” University of Medicine and Pharmacy, 16 Universităţii Street, 700115 Iaşi, Romania; rusu.i.gabriela@umfiasi.ro; 4 Department of Emergency Medicine, “Grigore T. Popa” University of Medicine and Pharmacy, 16 Universităţii Street, 700115 Iaşi, Romania; anca.haisan@umfiasi.ro; 5 Department of Surgery, “Grigore T. Popa” University of Medicine and Pharmacy, 16 Universităţii Street, 700115 Iaşi, Romania; smaranda-ioana.lupusoru@d.umfiasi.ro; 6 Department of Immunology, “Grigore T. Popa” University of Medicine and Pharmacy, 16 Universităţii Street, 700115 Iaşi, Romania; dconstantinescu_ro@yahoo.com; 7 Oftaprof Ophthalmology Clinic, 54 Stejar Street, 700327 Iaşi, Romania

**Keywords:** aqueous humor, cytokine biomarker, non-proliferative diabetic retinopathy, VEGF

## Abstract

*Background and Objectives*: Cytokines are cell-signaling proteins whose identification may serve as inflammatory markers or early indicators for progressive disease. The aim of our study was to quantify several cytokines in aqueous humor (AH) and their correlations with biochemical parameters in diabetic eyes with non-proliferative diabetic retinopathy (NPDR). *Materials and Methods*: A total of 62 eyes from 62 patients were included in the study: 37 eyes from nondiabetic patients (group 1), 13 diabetic eyes with no retinopathy changes (group 2) and 12 diabetic eyes with early and moderate NPDR (group 3). AH samples were collected during uneventful cataract surgery. The cytokines IL-1β, IL-6, IL-8, IL-10, IL-12, IP-10, MCP-1, TNF-α and VEGF were quantified using multiplex bead-based immunoassay. Due to unreliable results, IL-1β, TNF-α, IL-10 and IL-12 were excluded. Concentrations were compared between groups. Biochemical parameters (fasting blood sugar, glycated hemoglobin, C-reactive protein) and the duration of diabetes were recorded. *Results*: VEGF levels were significantly different between groups (*p* = 0.001), while levels of IL-6, IL-8, IP-10 and MCP-1 were comparable across all groups (*p* > 0.05). IL-6 concentration correlated with VEGF in group 1 (*rho* = 0.651, *p* = 0.003) and group 3 (*rho* = 0.857, *p* = 0.007); no correlation could be proved between IL-6, IL-8, IP-10, MCP-1 or VEGF and biochemical parameters. Duration of diabetes was not correlated with the cytokine levels in groups 2 and 3. The receiver operating characteristic (ROC) curve revealed that VEGF concentrations could discriminate early and moderate NPDR from diabetes, with an area under the curve (AUC) of 0.897 (*p* = 0.001, 95% CI = 0.74–1.0). *Conclusions*: Diabetes mellitus induces significant intraocular changes in the VEGF expression in diabetic patients vs. normal subjects, even before proliferative complications appear. VEGF was increasingly expressed once the diabetes progressed from no retinopathy to early or moderate retinopathy.

## 1. Introduction

Diabetic retinopathy (DR) is a chronic inflammatory neurovascular complication linked to diabetes mellitus (DM) [1]. DR progresses slowly and often shows no warning signs until advanced stages of the disease, when visual function can be severely impaired.

Cytokines are small cell-signaling proteins whose identification may serve as inflammatory biomarkers or early indicators of progressive disease [2,3]. Cytokines can be harvested from serum, tears, aqueous humor (AH) or vitreous humor (VH) [4,5]. It has been shown that concentrations from AH and VH are comparable [6,7], while the serum quantifications might not directly reflect the intraocular changes. For these findings, ocular immune privilege and blood–ocular barrier were incriminated [8,9]. In this respect, the quantification of such pro-inflammatory cytokines from AH and/or VH is more relevant for ocular pathologies with an inflammatory component such as DR [10].

Chronic hyperglycemia in diabetic patients enhances cellular oxidative stress, vascular endothelial dysfunction with loss of pericytes and alterations of the retinal blood flow via endothelin-1 mediation [11,12,13]. Local induced ischemia and inflammation trigger the up-regulation and synthesis of the abnormal release of growth factors that facilitate retinal angiogenesis and neovascularization, found in DR. Regardless of the DR stage, low-grade chronic inflammation has been described in all DR categories [14]. Unfortunately, despite this finding, current treatment options address only the advanced stages of the disease. Early and moderate stages are monitored according to existing protocols, but no marker has been identified in relation to the risk of conversion to advanced or proliferative forms. Detecting potential biomarkers of the subclinical or early stages of DR could result in further insights into DR management [1,15] and orient toward the risks of developing complications related to DR.

The aim of our study was to compare the AH inflammatory profile in patients with DM that was associated or not with early/moderate changes of non-proliferative diabetic retinopathy (NPDR). Levels of several pro-inflammatory cytokines were compared with normal subjects, keeping in mind a potential identification of specific molecules that might serve as diagnostic value or as a predictor of the risk of conversion to advanced/neovascular forms of DR.

## 2. Materials and Methods

### 2.1. Study Subjects

This comparative cross-sectional study included 62 eyes from 62 patients who underwent uncomplicated cataract surgery in the Department of Ophthalmology within Saint Spiridon Clinical Emergency Hospital, Iaşi, Romania, between August 2017 and August 2018. The patients were grouped in 3 categories: (1) 37 eyes from 37 healthy patients (group 1), (2) 13 eyes from 13 patients with DM without DR (group 2) and (3) 12 eyes from 12 patients with DM and NPDR without diabetic macular edema (DME) (group 3). We included in the study only cases with previously diagnosed DM. The study was conducted in accordance with the Declaration of Helsinki, and the protocol was approved by the Ethics Committee of Grigore T. Popa University of Medicine and Pharmacy (No. 13948/13 July 2017). All subjects signed an informed consent upon inclusion in the study.

### 2.2. Preoperative Evaluation

All patients underwent a complete ophthalmological examination that included best corrected visual acuity (BCVA, Snellen chart), intraocular pressure (IOP, mmHg) by Goldmann tonometer, anterior segment biomicroscopy and dilated fundus examination. Demographic data (age, sex) were collected, and fasting blood sugar (mmol/L), glycated hemoglobin (%) and C-reactive protein (CRP, mg/L) were recorded in all 62 patients. Duration of diabetes (years) and anti-diabetic treatment were recorded for the diabetic patients.

Patients with history of trauma, previous ocular surgeries, glaucoma, intraocular hypertension, uveitis, age-related degeneration, retinal vascular occlusions or systemic autoimmune or inflammatory diseases were excluded.

### 2.3. Surgical Procedure

All cataract surgeries were performed by a single experienced surgeon (D.C.) under topical anesthesia. Volumes of 0.1–0.2 mL of AH were harvested with a 30G tuberculin syringe via the first paracentesis that allowed access into the anterior chamber before uneventful phacoemulsification. Samples were immediately transferred into Eppendorf tubes (*Eppendorf* Tubes^®^ *3810X*, Hamburg, Germany) and stored at −80 °C until final analysis.

### 2.4. Aqueous Humor Analysis

Nine cytokines were quantified using the undiluted AH samples: interleukin-1β (IL-1β), interleukin-6 (IL-6), chemokine (CXC motif) ligand 8 (CXCL8/IL-8), interleukin-10 (IL-10), interleukin-12 (IL-12), chemokine (CXC motif) ligand 10 (CXCL10/IP-10), chemokine (CC motif) ligand 2/monocyte chemoattractant protein-1 (CCL-2/MCP-1), tumor necrosis factor α (TNF-α) and vascular endothelial growth factor (VEGF). The concentrations of the cytokines were measured by cytometric bead array with a Luminex polystyrene color bead-based multiplex assay (Luminex Screening Human Assay, LXSAH-09, RnD Systems). The assay was conducted according to the manufacturerʼs instructions.

Results (pg/mL) were automatically expressed as outputs that provided: (1) left-censored cytokine concentrations where the concentrations were below the limit of detection (<LOD) and the true value was unknown (“lost” data), (2) concentrations in range (CR) of valid standards where the concentrations were estimated accurately (the interval between the lower limit of quantification, LLOQ, and the upper limit of quantification, ULOQ) and (3) cytokine concentrations falling outside the LLOQ–ULOQ interval (extrapolated data).

### 2.5. Statistical Analysis

Statistical analysis in our study used SPSS^®^ 28 (Armonk, NY, USA: IBM Corp). Data were recorded as mean ± SD (standard deviation). For the cytokine concentrations, the median ± range were also recorded. The Shapiro–Wilk test was used for testing the normality. The Kruskal–Wallis test was used for differences in age, BCVA and CRP and cytokine levels between groups; if the test result was significant, a post hoc analysis was automatically displayed for pairwise comparisons. Fisher exact test was used for differences in sex between groups. The Mann–Whitney U test was used for differences in duration of DM. One-way analysis of variance test (ANOVA) was used for differences in IOP, fasting blood sugar and HbA1c values between groups; if the test was significant, a Bonferroni post hoc analysis was used for pairwise comparisons. Spearman’s correlation test was used for testing correlations between cytokine concentration and the demographic, clinical and biochemical characteristics of the patients. Multiple regression analysis was further employed when correlations between variables were statistically significant. The strength of the effect of each individual independent variable on the dependent variable was measured with the beta coefficient (β). To identify potential diagnostic biomarkers, the receiver operating characteristic (ROC) curve was used to evaluate the performance of cytokine concentrations in distinguishing between the groups. The area under the curve (AUC) was calculated. A *p* value less than 0.05 was considered statistically significant.

The sample size was calculated a priori using G*Power 3.1 and was estimated to be *n* = 12 per group, with a power of 80% and an alpha level of 0.05. The total sample size (62 patients) reflects the number of samples collected over a one-year period considering the inclusion criteria. In our study, we included all the patients from whom we harvested the AH knowing that it was possible that we might lose some cases after conducting the assay. Although the three groups were not equal, we managed to preserve the power at this level of the study, as we did not recruit more than four controls to one case.

## 3. Results

### 3.1. Characteristics of the Participants

The demographic, clinical and biochemical characteristics of the three groups are presented in Table 1.

In group 3 with DM and NPDR changes (early/moderate), we identified the youngest patients among the three study groups (*p* = 0.042), with significantly higher IOP (*p* = 0.016) and poorer glycemic control (*p* = 0.001). Sex distribution was comparable between groups. Duration of diabetes was similar between groups 2 and 3, while glycemic control was poorer in the eyes that had already developed any sign of retinopathy vs. the eyes from diabetic patients with DR changes. Despite this finding, the inflammatory status was statistically comparable between the two diabetic groups regardless of the presence or absence of DR; as expected when compared with the control group, both group 2 and group 3 had higher levels of CRP (group 1 vs. group 3, *p* = 0.001 and group 1 vs. group 2, *p* = 0.046). In our study, we observed that more patients in group 3 were using insulin to control their blood sugar (9/12 subjects), while the remaining (3/12 subjects) were on oral medications (OADs). The opposite situation was found in the group with DM who did not have DR as a complication, meaning that the majority were using OADs as diabetes treatment (10/13 subjects) and that only 3/10 subjects had started insulin-based medication.

### 3.2. Cytokine Analysis

In our study, with the Luminex Screening Human Assay LXSAH-09 from RnD Systems, the LLOQs were 16.22 pg/mL for IL-1β, 4.86 pg/mL for IL-6, 4.72 pg/mL for IL-8, 3.68 pg/mL for IL-10, 132.17 pg/mL for IL-12, 2.79 pg/mL for IP-10, 34 pg/mL for MCP-1, 9.04 pg/mL for TNF-α and 9.02 pg/mL for VEGF.

Out of the 62 analyzed AH samples, the concentrations for IL-1β and TNF-α were below LOD for 98.38% of cases and 53.22%, respectively, and were excluded from final analysis. One case had a concentration below LOD for IL-6, and three cases had concentrations below LOD for IL-2. These cases were also excluded from the final analysis.

After analyzing the detected and quantified concentrations of all AH cytokines, we concluded that all extrapolated data were for concentrations under the LLOQ. None of the samples had extrapolated data greater than the ULOQ. MCP-1 was the only AH cytokine detected and quantified within the range of valid standard for all patients in all three groups. We excluded from the final analysis all the extrapolated data; comparisons and correlation were made only for the concentrations within the range of a valid standard. For IL-10 and IL-12 we could not run any statistical analysis between groups due to the small number of patients in each group. A summary of cytokine analysis for group 1 is presented in Table 2.

A summary of cytokine analysis for group 2 is presented in Table 3.

A summary of cytokine analysis for group 3 is presented in Table 4.

**Figure 1:**
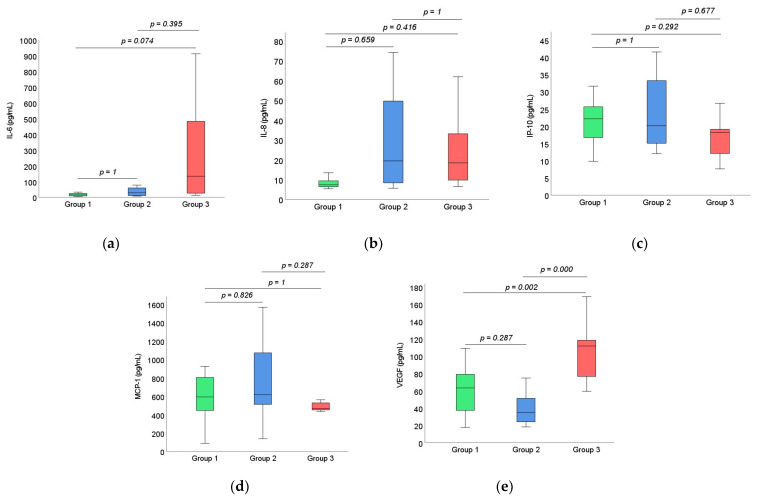
Cytokine concentrations for the three groups: (**a**) IL-6; (**b**) IL-8; (**c**) IP-10; (**d**) MCP-1; (**e**) VEGF. IL-6: interleukin-6; IL-8: interleukin-8; IP-10: chemokine ligand 10; MCP-1: monocyte chemoattractant protein-1; VEGF: vascular endothelial growth factor.

Figure 1 illustrates the comparison of IL-6, IL-8, IP-10, MCP-1 and VEGF concentrations between the study groups; no significant differences could be found (*p* > 0.05) in our set of patients in the above-mentioned cytokines except for VEGF (*p* = 0.001).

**Figure 2:**
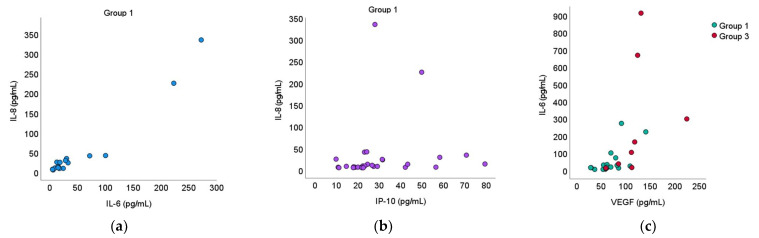
Cytokine correlations (**a**) IL-8 with IL-6; (**b**) IL-8 with IP-10; (**c**) IL-6 with VEGF.

Further analysis revealed that in group 1, IL-8 concentrations had significant positive correlations with IL-6 (*rho* = 0.847, *p* = 0.001) and IP-10 (*rho* = 0.493, *p* = 0.007) concentrations. Concentrations of IL-6 and VEGF were significantly positively correlated in both group 1 (*rho* = 0.651, *p* = 0.003) and group 3 (*rho* = 0.857, *p* = 0.007) (Figure 2).

**Figure 3:**
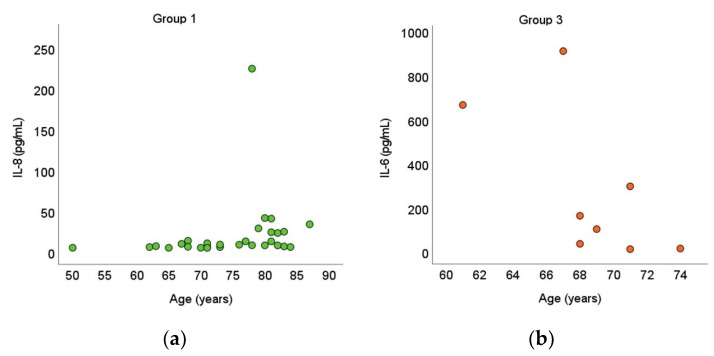
Cytokine correlations with age (**a**) IL-8; (**b**) IL-6.

IL-8 concentration had a significant positive correlation with age in group 1 (*rho* = 0.384, *p* = 0.036), and IL-6 concentration had a significant negative correlation with age in group 3 (*rho* = −0.711, *p* = 0.048) (Figure 3).

There were no significant correlations of IL-6, IL-8, IP-10, MCP-1, and VEGF concentrations with IOP, fasting blood sugar, HbA1c or CRP in the studied groups (*p* > 0.05).

For patients in group 2 and group 3, there were no correlations of IL-6, IL-8, IP-10, MCP-1 or VEGF concentrations with DM duration (*p* > 0.05).

Multiple regression analysis identified IL-6 concentration as a significant predictor (β = 0.917, *p* = 0.001) of IL-8 concentration (*R*^2^ = 0.952, *p* = 0.001) in group 1, while age (β = −0.117, *p* = 0.116) and IP-10 (β = 0.051, *p* = 0.439) had no significant influence.

In multiple regression analysis, neither age (β = −0.658, *p* = 0.08) nor VEGF (β = 0.361, *p* = 0.282) reached statistical significance as predictors of IL-6 concentration (*R*^2^ = 0.551, *p* = 0.135) in group 3.

**Figure 4:**
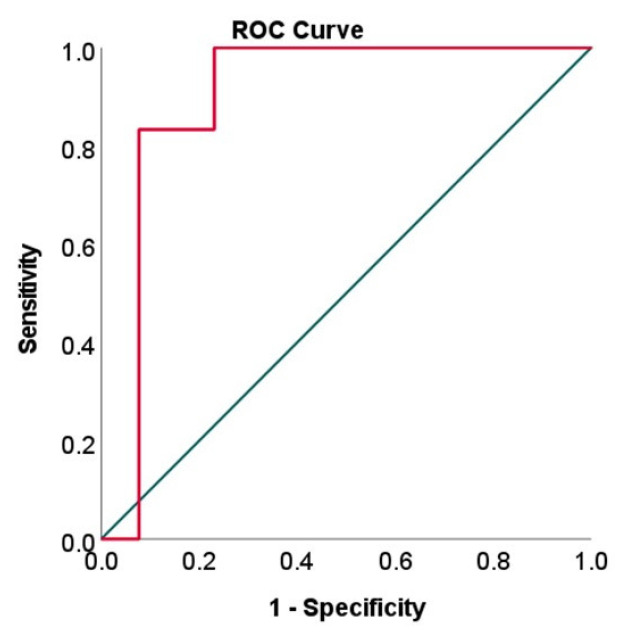
Receiver operating characteristic (ROC) curve to evaluate VEGF to differentiate DM from DM and NPRD.

The receiver operating characteristic (ROC) curve analysis revealed that VEGF concentrations differentiated early and moderate NPDR from DM patients with an area under the curve (AUC) of 0.897 (*p* = 0.001, 95% CI = 0.74–1.0) (Figure 4).

At a VEGF concentration cut-off value of 75.79 pg/mL, the sensibility (true positive) was 83.3%, and the 1-specificity (false positive) rate was 7.7% (the lowest rate among the false positives). At a VEGF concentration cut-off value of 63.71 pg/mL, the sensibility (true positive) was 91.7% (the highest rate of true positive), but the 1-specificity (false positive) increased to 23%.

## 4. Discussion

DR is the most frequent ocular complication of DM and a major cause of blindness through neovascularization (proliferative retinopathy) and DME [16].

Many studies highlighted that the actions of multiple mediators combine in the pathogenesis of DR. In the present study, we compared the inflammatory profiles via different cytokines in the AH of nondiabetic vs. diabetic patients using cytometric bead array (CBA). Such methods exhibit certain advantages over conventional enzyme-linked immunosorbent assay (ELISA) techniques because of the simultaneous detection of multiple analytes and the quantification in small sample volumes with higher repeatability and sensitivity [17,18].

IL-1β induces vascular dysfunction with increased vascular permeability [19]. Studies found correlations between IL-1β concentrations in serum and AH and the severity of DR. In our study, IL-1β was below LOD, so we could not analyze its contribution to the inflammatory profiles of our subjects. IL-12 is a pro-inflammatory cytokine, and high AH levels were detected in non-treated DR compared with patients with treated DR and patients without DR, suggesting a local secretion [20]. Because most of its quantifications in our study were below LOD, we could not confirm the increased local production mentioned above.

IL-10 is an anti-inflammatory and anti-angiogenic cytokine that down-regulates the production of pro-inflammatory cytokines [21]. IL-10 concentrations were different among different studies. In our study, IL-10 was excluded from the final analysis because of the number of extrapolated values. However, Zhang and et. found that IL-10 concentrations were higher in nondiabetic patients than in diabetic patients. Moreover, IL-10 concentrations had a significant negative correlation with DR severity [22]. Cheung et al. found that IL-10 concentrations were lower in DR and DM groups than in nondiabetic patients. The rate of detection of IL-10 was also lower in patients with DM than in controls [23]. On the contrary, Chen et al. found similar IL-10 concentrations in nondiabetic patients, patients with DM and patients with DM and DR [24]. Wu et al. found that IL-10 increased gradually with DR severity [17].

IL-6 is a potent pro-inflammatory cytokine up-regulated early in inflammation [19], and it is involved in inflammation and angiogenesis by VEGF induction [5]. IL-6 and VEGF AH concentrations are correlated [25]. Moreover, AH and VH levels correlate with DR severity and with the level of proteins [6]. The role of IL-6 in inflammation and pathophysiology of DR was also indicated by Chen et al. who found that high levels of IL-6, its soluble receptor (sIL-6R) and soluble gp130 (sgp130) in both serum and AH in patients with DR [26] are associated with longer DM duration, fasting blood sugar and HbA1c. In our study, there were no correlations between IL-6 and DM duration, fasting blood sugar or HbA1c.

IL-8 is a pro-inflammatory cytokine produced by the ischemic retina. Since IL-8 concentrations showed positive correlations with the severity of DME, IL-8 might be involved in the development of DME [27]. Feng et al. studied the levels of IL-1β, IL-6, IL-8 and TNF-α in the AH of patients with DR and found higher levels in both the 5-year and 10-year DR groups compared with the 5-year and 10-year DM groups [28].

IP-10 concentrations showed a positive correlation with VEGF when analyzed in vitreous [29], but no correlation was found when assessing AH [24]. We also found no correlation between IP-10 and VEGF. In our study, IL-8 correlated with IP-10 in nondiabetic patients, but no correlation was found in DM and DR groups.

MCP-1 is involved in leukostasis and attracting macrophages into areas with low perfusion [30], and high levels of both AH and VH are correlated with DME [27]. Serum MCP-1 has been suggested as a potential biomarker of DR in patients with early-onset type 2 DM [31]. Chen et al. found no significant differences in AH MCP-1 concentrations between patients with DR and patients without DM [24]. On the contrary, Tashimo et al. studied macrophage migration inhibitory factor (MIF) and MCP-1 concentrations in AH and found that the two correlated with each other and both correlated with the DR severity [32]. In our study, there were no statistical differences between DR patients and DM and nondiabetic patients.

In a review and meta-analysis about the role of TNF-α in DR, Yao et al. reported significant differences between DR and nondiabetic patients with respect to TNF-α concentrations, with higher values in DR patients [33]. Loukovaara et al. found no difference between PDR and NPDR [34], while Chen et al. reported lower concentrations in DR patients than in nondiabetic patients [24]. In our study, TNF-α was below the LOD for nondiabetic patients, DM and NPDR.

In the present study, VEGF was consistent with DM and DM complicated with DR status. VEGF is a pro-inflammatory molecule with a key role in neovascularization development and abnormally high vascular permeability [35]. VEGF has an important role both in NPDR and in PDR, and the beneficial role of anti-VEGF therapy for NPDR with DME is well-known. Recent studies have investigated NPDR without DME management, and there is a debate between conventional management derived from the Early Treatment Diabetic Retinopathy Study (ETDRS), which included controlling the systemic conditions and observation, and anti-VEGF therapy. The PANORAMA and Protocol W randomized trials showed that an early intervention with aflibercept in moderately severe to severe NPDR significantly improved DR severity scale scores and reduced vision-threatening complications of DR [36,37,38,39].

Previous studies proposed serum VEGF level as a biomarker for subclinical DR [40]. However, these included in the analysis also patients with PDR. In our study, we assessed a selected panel of cytokines in nondiabetic patients, patients with DM without DR and patients with NPDR without DME. Moreover, the NPDR group in our study included only patients with early and moderate NPDR. VEGF concentrations showed similar values in the nondiabetic and in DM groups. Significant differences were spotted between DM and NPDR and between the nondiabetic group and NPDR. Our focus was on determining if aqueous humor VEGF concentrations could have diagnostic value for NPDR. The AUC was 0.897. Although AH cytokine assessment is an invasive method, it could be used in selected patients at the same time as otherintraocular interventions so that the burden is eased for the patient. Patients undergoing cataract surgery could benefit from AH screening for diagnostic biomarkers for early DR, and the results could be further interpreted for predictive and prognostic value.

In our study, we found a significant difference with respect to patients’ age, with the youngest patients in DM with NPDR. The mean difference between this group and the other two groups was around six years. The age difference could be explained either by denser cataracts in younger patients or by earlier presentation for cataract surgery in DM with NPDR. Studies have shown that aging is associated with para-inflammation in both physiological and pathological conditions. Tissular stress induced by oxidative stress acts as a local trigger for para-inflammation, blood–retinal barrier breakdown, glial activation and increased cytokines production, all of which are characteristics of age-related retinal para-inflammation [41].

Inflammatory and oxidative stress pathway cross-talk has an important role in DR pathogenesis. Studies have shown that inflammatory cytokines and oxidative stress-related pathways in different systems such as red blood cells and retinal pigment epithelium (RPE) cells also provide significant potential biomarkers for DM and DM and DR. Red blood cell membrane fluidity is influenced by reactive oxygen species. Recent studies indicated that a more fluid red blood cell membrane may represent a marker of DR in type 1 DM [42]. In an experimental model, Bianchetti et al. found that under induced hyperglycemia human RPE cells treated with ω3-polyunsaturated docosahexaenoic acid (DHA) did not result in cellular apoptosis, suggesting the counteracting effect of DHA on oxidative stress and apoptosis [43].

In our study, we found significantly higher values of HbA1c in NPDR compared with the control group and DM. Glycated hemoglobin constitutes an important parameter in the evaluation of long-term diabetes-related complications. Zhang et al. found that HbA1c values have good overall accuracy for DR diagnosis [44]. One important drawback of cytokine assessment in the intraocular fluids is that it is invasive. However, harvesting the concentrations of different cytokines from AH at the time of other intraocular surgery and structural changes analysis (retinal thickness, choroidal thickness) could bring information with respect to the future evolution of DR and could have predictive value for DR response to treatment.

The cross-sectional nature of the study and the analysis of a limited number of cytokines were limitations of our study. Excluding all the extrapolated data from the final analysis after conducting the assay, in order to have relevant comparisons and correlations, is another limitation of the study because of the small final sample size. Additionally, in our study, the patients were not age-matched, and knowing that aging is associated with increased levels of circulating and intraocular cytokines, this might be a limitation when comparing different groups of patients. Structural analysis and further correlation could not be performed because there were multiple cases with significant media opacities that prevented optimal optical coherence tomography image acquisition. The use of a screening assay for measuring the concentrations of cytokines in our study might be one possible cause of not detecting and quantifying all the selected cytokines. We hypothesize that using high-performance assays could overcome this drawback and increase the sensitivity for detecting the cytokines of interest. However, the screening test proved useful for identifying VEGF as a possible diagnostic biomarker for NPDR.

## 5. Conclusions

In conclusion, the results of our study revealed that DM induces significant intraocular changes in VEGF expression in diabetic patients vs. normal subjects, even before proliferative complications or DME appear. The VEGF levels were increasingly expressed once the diabetes progressed from no retinopathy to early/moderate retinopathy, so VEGF concentrations could have good diagnostic value for early DR. Extended studies are required to further demonstrate if VEGF could be used as a marker to predict how fast a conversion might occur toward neovascular complications based on a specific detected level or mathematically calculated trend.

## Figures and Tables

**Table 1:** Demographic, clinical and biochemical data for each study group.

Parameters	Group 1 (*n* = 37)	Group 2 (*n* = 13)	Group 3 (*n* = 12)	*p* ^1^
Age (y)	72.78 ± 9.92	72.08 ± 6.87	66.42 ± 5.14	0.042
Sex (*n*, male:female)	17:20	5:8	4:8	0.714
BCVA (decimal scale)	0.23 ± 0.24	0.10 ± 0.19	0.20 ± 0.19	0.222
IOP (mmHg)	12.84 ± 2.66	13.77 ± 2.48	15.5 ± 3.03	0.016
Duration of DM (y)	–	6.83 ± 5.13	10 ± 7.9	0.406
Fasting blood sugar (mmol/L)	5.49 ± 0.67	7.39 ± 1.55	10.26 ± 3.54	0.001
HbA1c (%)	5.5 ± 0.41	6.78 ± 0.84	8.49 ± 2.63	0.001
CRP (mg/L)	2.56 ± 2.33	8.85 ± 6.33	6.95 ± 8.55	0.001
DM treatment (*n*, OADs:Insulin)	–	10:3	3:9	–

^1^ Significance of differences between groups. BCVA: best corrected visual acuity (Snellen chart); IOP: intraocular pressure; HbA1c: glycated hemoglobin; CRP: C-reactive protein; OADs: oral antidiabetic drugs.

**Table 2:** Summary of cytokine evaluation in group 1 (*n* = 37).

Cytokine	<LOD (*n*, %)	<LLOQ (*n*, %)	Valid Cases (*n*)	Observed Concentrations (OC) (pg/mL)	Concentration in Range (CR) (pg/mL)	Mean of CR (pg/mL)	Median of CR (pg/mL)
IL-1β	36 (97.29)	1 (2.7)	0	14.27	–	–	–
IL-6	1 (2.7)	18 (48.64)	18	0.31–272.15	5.27–272.15	50.30	18.11
IL-8	0 (0)	7 (18.91)	30	2.79–334.51	5.35–334.51	31.86	9.36
IL-10	0 (0)	33 (89.18)	4	0.64–5.15	3.90–5.15	4.44	4.37
IL-12	2 (5,4)	30 (81.08)	5	0.05–332.54	153.17–332.54	234.44	229.35
IP-10	0 (0)	1 (2.7)	36	3.13–79.49	9.87–79.49	28.76	23.75
MCP-1	0 (0)	0 (0)	37	150.57–4825.60	150.57–4825.60	812.06	603.29
TNF-α	26 (70.27)	11 (29.72)	0	0.04–6.71	–	–	–
VEGF	0 (0)	1 (2.7)	36	6.83–141.09	17.73–141.09	63.49	63.39

The symbol – means that all values were outside the valid range or there was only one value in the valid range. IL-1β: interleukin-1β; IL-6: interleukin-6; IL-8: interleukin-8; IL-10: interleukin-10; IL-12: interleukin-12; IP-10: chemokine ligand 10; MCP-1: monocyte chemoattractant protein-1; TNF-α: tumor necrosis factor-α; VEGF: vascular endothelial growth factor; LOD: limit of detection; LLOQ: lower limit of quantification; ULOQ: upper limit of quantification; cytokine concentrations <LOD–left-censored data; <LLOQ–below LLOQ; Valid cases = number of cases included in the final statistical analysis; OC: observed concentrations = extrapolated data (<LLOQ–UPLQ>); CR: concentration in range = quantified concentrations that could actually be used in statistical analysis that were within the range of valid standards (LLOQ–ULOQ).

**Table 3:** Summary of cytokine evaluation in group 2 (*n* = 13).

Cytokine	<LOD (*n*, %)	<LLOQ (*n*, %)	Valid Cases (*n*)	Observed Concentrations (OC) (pg/mL)	Concentration in Range (CR) (pg/mL)	Mean of CR (pg/mL)	Median of CR (pg/mL)
IL-1β	13 (100)	0 (0)	0	–	–	–	–
IL-6	0 (0)	9 (69.23)	4	0.49–77.19	7.32–77.19	35.21	28.16
IL-8	0 (0)	3 (23.07)	10	1.86–74.11	5.62–74.11	28.35	19.42
IL-10	0 (0)	13 (100)	0	1.00–3.81	–	–	–
IL-12	1 (7.69)	11 (84.61)	1	0.05–153.17	–	–	–
IP-10	0 (0)	0 (0)	13	12.04–85.89	12.04–85.89	28.71	20.55
MCP-1	0 (0)	0 (0)	13	89.83–1391.52	89.83–1391.52	652.69	589.60
TNF-α	9 (69.23)	4 (30.76)	0	0.63–1.63	–	–	–
VEGF	0 (0)	0 (0)	13	18.28–1190.09	18.28–1190.09	130.55	45.21

**Table 4:** Summary of cytokine evaluation in group 3 (*n* = 12).

Cytokine	<LOD (*n*, %)	<LLOQ (*n*, %)	Valid Cases (*n*)	Observed Concentrations (OC) (pg/mL)	Concentration in Range (CR) (pg/mL)	Mean of CR (pg/mL)	Median of CR (pg/mL)
IL-1β	12 (100)	0 (0)	0	–	–	–	–
IL-6	0 (0)	4 (33.33)	8	0.68–911.81	12.96–911.81	276.36	134.08
IL-8	0 (0)	2 (16.66)	10	3.69–109.65	6.53–109.65	31.52	19.91
IL-10	0 (0)	11 (91.66)	1	0.76–10.72	–	–	–
IL-12	0 (0)	8 (66.66)	4	0.05–264.90	132.50–264.90	185.71	172.72
IP-10	0 (0)	1 (8.33)	11	2.32–101.04	7.65–101.04	25.42	18.27
MCP-1	0 (0)	0 (0)	12	139.24–2040.32	139.24–2040.32	829.13	550.03
TNF-α	7 (58.33)	5 (41.66)	0	0.08–5.10	–	–	–
VEGF	0 (0)	0 (0)	12	59.51–506.60	59.51–506.60	149.01	115.51

## Data Availability

The datasets used and/or analyzed during the present study are available on request from the corresponding author.
